# Using roaming behaviours of dogs to estimate contact rates: the predicted effect on rabies spread

**DOI:** 10.1017/S0950268819000189

**Published:** 2019-03-05

**Authors:** Emily G. Hudson, Victoria J. Brookes, Michael P. Ward, Salome Dürr

**Affiliations:** 1 Sydney School of Veterinary Science, The University of Sydney, Camden, Australia; 2 Veterinary Public Health Institute, University of Bern, Liebefeld, Switzerland

**Keywords:** Contact estimation, disease modelling, free-roaming dogs, heterogeneous contacts

## Abstract

Domestic dogs display complex roaming behaviours, which need to be captured to more realistically model the spread of rabies. We have previously shown that roaming behaviours of domestic dogs can be categorised as *stay-at-home*, *roamer* and *explorer* in the Northern Peninsular Area (NPA), Queensland, Australia. These roaming behaviours are likely to cause heterogeneous contact rates that influence the speed or pattern of rabies spread in a dog population. The aim of this study was to define contact spatial kernels using the overlap of individual dog utilisation distributions to describe the daily probability of contact between pairs of dogs exhibiting these three *a priori* roaming behaviours. We further aimed to determine if the kernels lead to different predicted rabies outbreaks (outbreak duration and number of rabid dogs) by incorporating the spatial kernels into a previously developed rabies spread model for the NPA. Spatial kernels created with both dogs in a pair being *explorers* or one dog *explorer* and one dog *roamer* (who roamed away from their residence) produced short but large outbreaks compared with spatial kernels with at least one *stay-at-home* dog. Outputs from this model incorporating heterogeneous contacts demonstrate how roaming behaviours influence disease spread in domestic dog populations.

## Introduction

Rabies causes approximately 60 000 human deaths annually, 99% of which are caused by domestic dogs [1, 2]. A lack of preparedness can contribute to substantial dog deaths and some loss of human life when a rabies outbreak occurs in previously rabies-free regions; for example, in the 2008 rabies outbreak in Bali [3, 4]. The annual probability that at least one rabid dog from Indonesia enters north-west Cape York Peninsula, which includes the communities of the Northern Peninsula Area (NPA), Queensland, via an illegal fishing boat and infects a resident NPA dog has been estimated to be 8.3 × 10^−5^ (standard error 1.4 × 10^−4^) per year [5]. This probability is due to the prevalence of rabies in Indonesia and the proximity of north-west Cape York Peninsula to Indonesia facilitating boat travel between the countries [5]. Although this probability is small, the impact of a rabies outbreak in the NPA communities is likely to be high given the large population of free-roaming domestic dogs in these communities [6]. To help mitigate the risk of exotic disease spread, disease transmission models can be used to simulate outbreaks to investigate potential patterns of disease spread and effectiveness of different control strategies. For model predictions to be accurate, knowledge of the underlying process of disease spread is needed so that it can be represented in the model structure.

A key driver of infectious disease spread is how individuals contact each other. In models of canine rabies spread in Australia, contacts have been modelled in different ways. Rabies in wild dogs has been modelled using a stochastic transmission network (percolation) model in which the contact rate between two wild dogs is a function of the distance between the centroids within their home range (HR) and the dogs’ sociability [7]. The latter is defined as the dog's individual tendency to interact with other dogs, which is related to the daily area that the dog traverses [7]. Another model using ordinary differential equations within state transition models simulated rabies spread in three Australian dog populations; free-roaming island community domestic dogs, free-roaming peri-urban domestic dogs and wild dogs [8]. Finally, rabies outbreaks in northern Australian remote communities’ dog populations have been simulated by an agent-based model that uses a spatial kernel to describe the dog contacts [9]. This spatial kernel describes the daily probability of contact between two dogs as a function of the distance between their respective residences; the greater the distance between the residences, the less likely the dogs will make contact. These models all estimate contact rates based on some sort of contact field data from their target populations. However, our understanding of movements and interactions in the target populations is increasing and such knowledge can be used to update how contact rates are represented within existing infectious disease models.

Roaming behaviours of individual dogs are likely to influence interactions with other dogs and therefore, could influence the contact rate within the population and subsequently affect disease spread. A recent study conducted in the NPA using GPS datasets of 21 dogs described three roaming behaviours within the study population based on how the estimated utilisation distribution (UD) of each dog changed with increased durations of monitoring [10]. UDs have been used to describe roaming behaviours of various animals, including dogs [11–14]. It is the three-dimensional distribution that defines the probability of finding an animal at any location within its HR dependent on the relative temporal utilisation of the HR at that location. For example, the 50% and 95% isopleth define the areas where the animal spends 50% and 95% of their time, respectively. The three categories described in the NPA study were *explorer* dogs who often visited different places each day, *roamer* dogs whose core UD was around the owner's residence but made less frequent trips to different places than *explorers*, and *stay-at-home* dogs who spent most (if not all) of their time around their owner's residence [10]. These roaming categories could represent heterogeneity of contacts in the dog population of the NPA and could affect how rabies is spread within the population. For example, if all dogs were *explorer* dogs, we could expect that outbreaks would propagate faster due to their large HRs and expected high contact rates with other dogs. In simulation models, different spatial kernels describing each roaming category might be needed to accurately describe dog contacts and subsequent rabies spread.

Potentially diverse contact rates caused by the different roaming behaviours need to be estimated before they can be incorporated into the previously developed rabies model for the NPA [9] and their subsequent effects on rabies spread analysed. Previous studies in northern Australia, including the NPA, have estimated contacts by analysing GPS data of pairs of dogs to determine spatiotemporal proximity [9, 15, 16]. However, this same approach cannot be applied to the data collected in the study that established the different roaming behaviours because GPS fix recording intervals of 15 min were utilised [10], which is too long to infer likely contacts. A previous study found evidence of significant positive correlation (up to 0.82) between UD overlap and contact rate by placing proximity loggers on 15 racoons (55 and 66 dyads in summer and winter, respectively) and analysing the strength of the relationship between contact rate and UD overlap (given by various indices [17]) of the dyads [18]. Therefore, when such data that can directly estimate the number of contacts between dogs are lacking, UD overlap between two dogs could be used as a proxy for contacts and more specifically, given the spatial and temporal elements of a UD, the probability of contact could be estimated. The aim of this study was to define spatial contact kernels using the overlap of individual dog UDs to describe the daily probability of contact between pairs of dogs exhibiting the three categories of roaming behaviour previously described in the NPA dog population. We further aimed to determine if the kernels produced different rabies outbreaks (outbreak duration and number of rabid dogs) by incorporating the spatial kernels into the NPA model. The updated model can then be used to more accurately describe potential rabies outbreaks in the NPA and refine outbreak mitigation strategies.

## Methods

### Study area and data collection

The data used in this study were previously collected in the five communities of the NPA, Queensland (Bamaga, Injinoo, New Mapoon, Seisia and Umagico) and consists of GPS locations observed from owned domestic free-roaming dogs [10]. Briefly, GPS units were attached to dogs in 2014 and 2016, using regular dog collars. Datasets of 21 dogs (10 from 2014 and 11 from 2016) were of sufficient monitoring length (>5 days) to be further analysed. All communities were represented and of the 21 dogs, nine were female and 12 were male. The dogs were categorised into three different roaming groups (*stay-at-home n* = 9; 2:1 males to females, *roamer n* = 6; 1:2 males to females, or *explorer n* = 6, 2:1 males to females) based on how the estimated UD of each dog changed with increasing durations of monitoring. For more details refer to Hudson *et al*. [10]. The same 21 GPS datasets and their subsequent categorisations of roaming behaviours are used in this current study.

### Spatial kernel estimation

The overlap between two dogs’ UDs at varying distances between the dogs’ residences was used to estimate a probability of contact between multiple pairs of dogs, which were in turn used to create spatial kernels. Sampled contact probabilities from the resulting spatial kernels and a bite probability are used in the simulation model to describe the probability of effective contact required to transmit rabies dependent on the distance between the two dogs’ residences. The roaming categories assigned by Hudson *et al*. [10] were used to create six types of spatial kernels based on all possible combinations of categories between a pair of dogs; two *explorer* dogs (EE kernel), an *explorer* dog and a *roamer* dog (ER kernel), an *explorer* dog and a *stay-at-home* dog (ES kernel), two *roamer* dogs (RR kernel), a *stay-at-home* dog and a *roamer* dog (SR kernel) and two *stay-at-home* dogs (SS kernel).

**Fig. 1.**
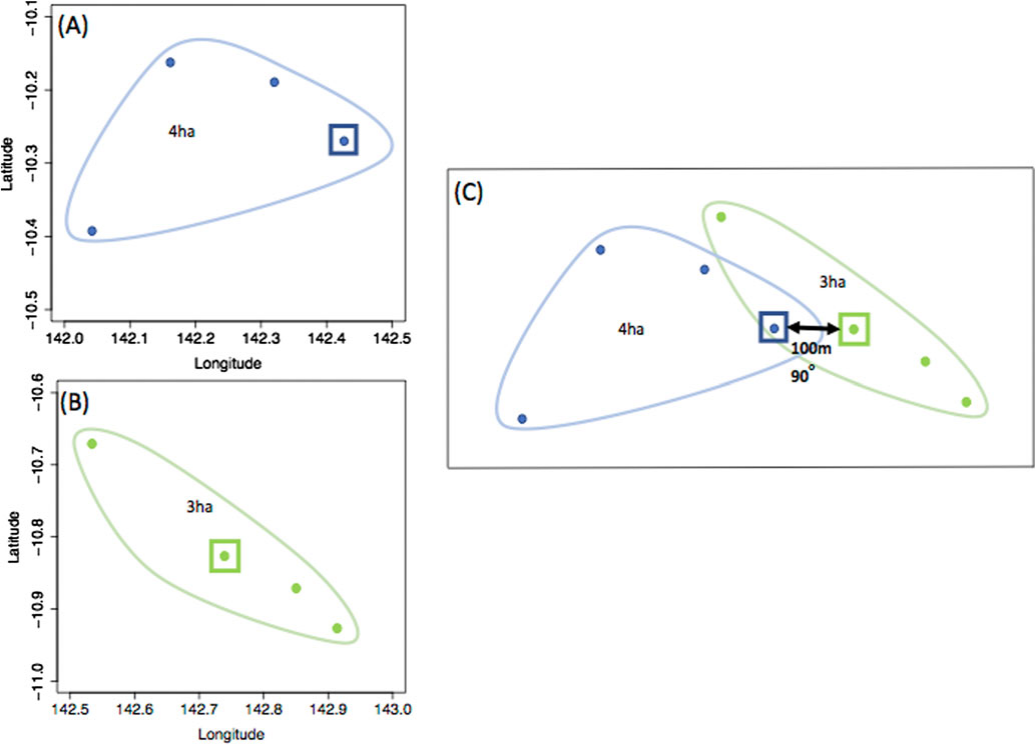
An example of dog GPS dataset translocation to simulate the probability of contact between a pair of dogs if their residences were 100 m apart in an east-west orientation. (a) dog *i*’s GPS dataset with the square representing the dog's residence, in which its home range (95% UD isopleth) is 4ha, (b) *j*’s GPS dataset with the square representing the dog's home in which its home range (95% UD isopleth) is 3ha, (c) the two dogs’ home ranges when their residence coordinates are changed to be 100 m apart in a 90^o^ direction. The home ranges – and UD to calculate the three-dimensional overlap – are unchanged, but translocated.

For each kernel type, all possible pairs of dogs (and their respective GPS datasets) were selected to build the respective kernels (e.g. two *explorer* dogs for the EE kernel; an *explorer* dog and a *stay-at-home* dog for the ES kernel). To estimate contact probability based on the UD overlaps between each pair of dogs in relation to the distance between their residences, the following procedure was implemented. First, the two dogs’ residences were placed at a location 10 m apart. Then, the two dogs’ 95% UDs were re-created around each simulated residence location based on the empirical GPS points’ bearings and distances from their original residence location (Fig. 1). The probability HR (PHR) overlap index was used to estimate the probability of concurrent roaming in the overlapping area of pairs of dogs’ UDs and calculated using the ‘adehabitatHR’ package in R [19, 20]. The PHR overlap index uses the overlap of two UDs to estimate the probability of finding dog *i* in dog *j*’s HR depending on dog *i*’s distribution of time in its own HR and *vice versa* (PHR_*j,i*_ and PHR_*i,j*_ respectively) [17]. The two probabilities given by the PHR index were multiplied to estimate the probability that both dog *i* and dog *j* are in the overlapping area based on respective proportion of time spent in the area. This probability was used as the probability of contact between dog *i* and dog *j.* The process of UD re-creation and calculation of the probability of contact was repeated for distances between the dog's residences from 10–1000 m by 10 m increments in a random direction (selected between 1–360°). Following simulation of UD overlap for all possible pairs of dogs within and between the roaming categories, the median contact probabilities and the 2.5% and 97.5% ranges at each distance were calculated for the six possible roaming combinations to create the six kernels and inform the variability of the kernels.

To predict the probability of contact for every 1 m between 10 and 1000 m, three-parameter logistic functions, four-parameter logistic functions and Weibull functions were fitted to each of the median, 2.5% and 97.5% range datasets via least squares methodology using the ‘minpack.lm’ R package [21] with parameter restrictions that limited the probability to values 0–1. The best fitting function for each dataset within each kernel (*n* = 18) was chosen based on the lowest extracted AIC value. The kernel creation was conducted on the University of Sydney's High Performance Computer using the R statistical program [20].

### Model simulations

#### The rabies spread model

In this study, we used a rabies spread model developed to predict rabies spread following an incursion in free-roaming domestic dog populations in northern Australia [9]. Several updates were made to the rabies spread model. The original model was based on a whole dog census in the NPA conducted in 2009 by local Animal Management Workers (AMWs); it concluded that there were 437 dogs in the NPA communities [9, 11]. A recent study re-estimated the NPA dog population using a survey of AMWs and local rangers and estimated the total population to be 813 dogs [6]. The number of dogs per household, the distance between each dog-owning household and the number of dogs in each community estimated were updated in the model using the information collected by Hudson *et al*. [6]. The housing density is uniform in the NPA communities because the houses are government-built and on equal sized land parcels. Based on the geolocations of dog-owning households in each community in Hudson *et al*. [6], the mean distance between dog-owning households in each community ranged between 209 m in Umagico and 530 m in Bamaga and the minimum distance between all dog-owning households in the NPA was 10 m. Two additional parameters were also incorporated. The first parameter defines the probability that an individual dog develops furious rabies (0.4) followed by a second parameter to model the increased bite probability after development of clinical rabies in a dog with the furious form of rabies (considered to be three times the probability of a bite by a non-clinical dog). These parameter estimates were based on previous studies [5] and authors’ assumptions.

#### Scenarios

Overall, 18 scenarios were simulated based on the six kernel types with three index dog scenarios each. The six kernel scenarios were simulated by incorporating each of the six spatial kernels into the model in turn. For example, only the EE kernel was used to inform contacts between all dogs for the EE scenarios and only the ER kernel was used to inform contacts between all dogs for the ER scenarios. The model used the kernel to determine the daily probability of contact between a pair of dogs based on the distance between their residences. The median, 2.5% and 97.5% percentile of the kernel corresponding to the distance between each dog's residence were used as the most likely, minimum and maximum, respectively, to create a *β*-PERT distribution in the model and the daily probability of contact for the pair of dogs sampled from this distribution. The spatial kernels only describe probability of contact between two dogs, and therefore, to estimate the probability of effective contact necessary for rabies transmission, the daily probability of contact was multiplied with a bite probability (dependent on the form of rabies of the infected dogs). This overall probability of effective contact is subsequently used to create a binomial distribution which is sampled to determine if effective contact was made between the two dogs in question. For all dogs that have been effectively contacted by an infected dog, a further rabies transmission probability is used to determine if the virus is transmitted following effective contact. For more details on the model refer to Dürr and Ward [9].

The three index dog scenarios were defined by the dog density of the NPA: (1) the index dog is randomly selected from the entire population, (2) the index dog is a fixed dog in a dog dense area (Bamaga), and (3) the index dog is a fixed dog in a dog sparse area (Seisia). Outbreak controls – for example, vaccination – were not implemented in the simulations. Summary statistics for the number of rabid dogs and outbreak duration were produced for the 18 scenarios. A Kruskal–Wallis test was used to identify statistically significant differences between outputs from the model scenarios and a post-hoc test (Dunn's Test with Bonferroni correction) was used to determine statistically significant pairwise differences (*P* < 0.01).

#### Simulations to achieve convergence of model outputs

The SS kernel scenario with a randomly selected index dog was used to determine the required number of simulations needed per scenario to achieve convergence of summary statistics. This scenario was selected for this exercise because it produced the greatest variance in model outputs. The coefficient of variation (CV) of model outputs has been suggested as a measurement to determine the number of simulations required to achieve convergence of model outputs [22]. The CV of the outbreak duration and number of rabid dogs was calculated for 10 sets of 100–4000 simulations. The smallest simulation number in which 97.5% of CVs in the previous 100 simulations were <0.04 (a useful measure of model output stability) for both number of rabid dogs and outbreak duration was chosen as the optimal number of simulations. The model simulations and statistical analyses were conducted on The University of Sydney's High Performance Computer using the R statistical program [20].

## Results

### Kernel creation

**Fig. 2.**
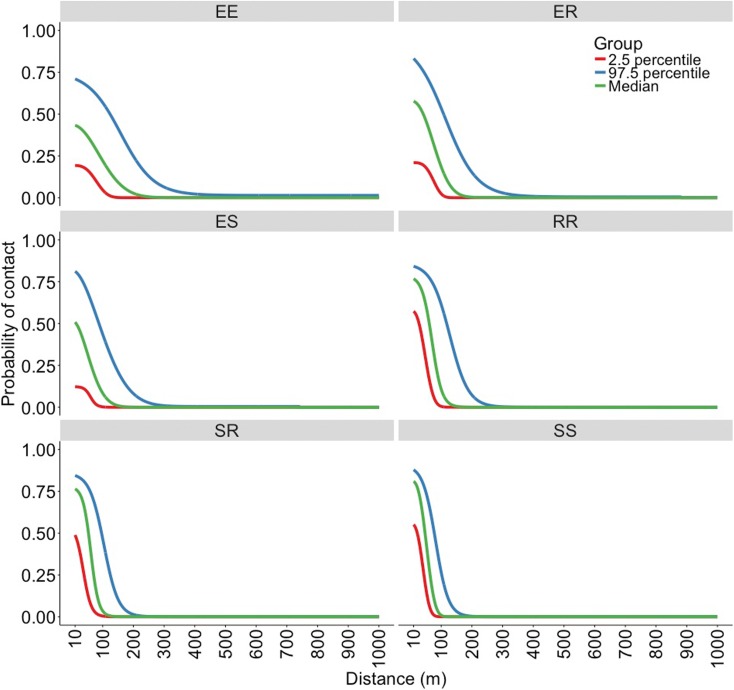
Spatial kernels produced in a simulation study based on all possible combinations of categories of roaming behaviour; two *explorer* dogs (EE kernel), an *explorer* dog and a *roamer* dog (ER kernel), an *explorer* dog and a *stay-at-home* dog (ES kernel), two *roamer* dogs (RR kernel), a *stay-at-home* dog and a *roamer* dog (SR kernel) and two *stay-at-home* dogs (SS kernel).

The best fitting functions and the parameters that were fitted to the overlap data to create the kernels are shown in Table 1. Visual representation of the six spatial kernels created from these functions for the different combinations of roaming categories are shown in Figure 2. The SS kernel had the greatest median contact probability at 10 m (0.809, 95% range = 0.551–0.878), and reached a probability of 0.1 and 0 at the shortest distances (78 and 141 m, respectively; Table 2). Conversely, the EE kernel had the lowest contact probability at 10 m (0.433, 95% range = 0.193–0.710) but reached 0.1 and 0 at the furthest distance (147 and 343 m, respectively; Table 2). The kernels without an *explorer* dog had the highest contact probabilities at shorter distances but also reached a zero probability at shorter distances compared with the kernels with an *explorer* dog. In contrast, the kernels with an *explorer* dog showed more variation in probabilities, creating wider ranges between 2.5% minimum and 97.5% maximum probability of contact (Table 2).

**Table 1.** Function parameters used to fit simulated utilisation distribution (UD) overlap data of overlapping dog utilisation distribution at incremental distances from 21 dog datasets collected in the Northern Peninsula Area, Queensland Australia

Kernel^a^	Function	Parameters
EE		
Median	Weibull (Asym, Drop, lrc, pwr)	Weibull (0.001303, −0.578108, −10.607419, 2.333159)
2.50%	Weibull (Asym, Drop, lrc, pwr)	Weibull (0, −0.2095, −17.1428, 3.8934)
97.50%	4-parameter logistic (A, B, xmid, scal)	Logistic (0.9413, 0.004717, 113.7, 51.24)
ER		
Median	Weibull (Asym, Drop, lrc, pwr)	Weibull (0, −0.4352, −9.7898, 2.0414)
2.50%	Weibull (Asym, Drop, lrc, pwr)	Weibull (0, −0.1929, −14.4917, 3.2238)
97.50%	4-parameter logistic (A, B, xmid, scal)	Logistic (0.75355, 0.01377, 158.81465, 53.71570)
ES		
Median	Weibull (Asym, Drop, lrc, pwr)	Weibull (0, −0.5189, −8.3546, 1.9440)
2.50%	3-parameter logistic (Asym, xmid, scal)	Logistic (0.1235, 60.3060, −10.0984)
97.50%	Weibull (Asym, Drop, lrc, pwr)	Weibull (0.004483, −0.813974, −9.184410, 1.895362)
RR		
Median	3-parameter logistic (Asym, xmid, scal)	Logistic (0.7863, 70.2314, −16.1642)
2.50%	Weibull (Asym, Drop, lrc, pwr)	Weibull (0.000481, −0.577691, −11.069992, 2.745385)
97.50%	3-parameter logistic (Asym, xmid, scal)	Logistic (0.8598, 127.8663, −30.3622)
SR		
Median	3-parameter logistic (Asym, xmid, scal)	Logistic (0.7776, 61.2093, −12.6910)
2.50%	3-parameter logistic (Asym, xmid, scal)	Logistic (0.5502, 36.8422, −12.9916)
97.50%	3-parameter logistic (Asym, xmid, scal)	Logistic (0.8591, 104.0302, −23.3257)
SS		
Median	Weibull (Asym, Drop, lrc, pwr)	Weibull (0, −0.8131, −12.2370, 2.9838)
2.50%	Weibull (Asym, Drop, lrc, pwr)	Weibull (0, −0.5567, −11.2435, 2.9152)
97.50%	3-parameter logistic (Asym, xmid, scal)	Logistic (0.9055, 81.8654, −20.6504)

Parameter Abbreviations from R [20]: Asym, a numeric parameter representing the asymptote; xmid, a numeric parameter representing the *x* value at the inflection point of the curve; scal, a numeric scale parameter on the input axis; A, a numeric parameter representing the horizontal asymptote on the left side; B, a numeric parameter representing the horizontal asymptote on the right side; Drop, a numeric parameter representing the change from Asym to the *y* intercept; lrc, a numeric parameter representing the natural logarithm of the rate constant; pwr, a numeric parameter representing the power to which *x* is raised.

a Spatial kernels based on all possible combinations of categories between a pair of dogs; two *explorer* dogs (EE kernel), an *explorer* dog and a *roamer* dog (ER kernel), an *explorer* dog and a *stay-at-home* dog (ES kernel), two *roamer* dogs (RR kernel), a *stay-at-home* dog and a *roamer* dog (SR kernel) and two *stay-at-home* dogs (SS kernel).

**Table 2.** Contact probabilities at example distances and distance to produce example contact probabilities produced by the six different spatial kernels based on different roaming categories to inform contact rates between a pair of dogs in the Northern Peninsula Area, Australia

Kernel^a^	Median contact Pr. at 10 m (95% range)	Median contact Pr. at 200 m (95% range)	Distance for median to reach Pr. = 0.1 (95% range)	Distance for median to reach Pr. = 0 (95% range)
EE	0.433 (0.193–0.710)	0.027 (0.000–0.248)	147 m (79–268 m)	343 m (211–1000 m)
ER	0.576 (0.210–0.832)	0.003 (0.000–0.151)	121 m (76–226 m)	311 m (181–881 m)
ES	0.508 (0.123–0.812)	0.002 (0.000–0.081)	96 m (46–191 m)	241 m (141–741 m)
RR	0.768 (0.573–0.842)	0.000 (0.000–0.073)	102 m (70–190 m)	261 m (151–721 m)
SR	0.764 (0.488–0.844)	0.000 (0.000–0.014)	86 m (57–152 m)	191 m (121–361 m)
SS	0.809 (0.551–0.878)	0.000 (0.000–0.003)	78 m (57–125 m)	141 m (91–271 m)

a Spatial kernels based on all possible combinations of categories between a pair of dogs: two *explorer* dogs (EE kernel), an *explorer* dog and a *roamer* dog (ER kernel), an *explorer* dog and a *stay-at-home* dog (ES kernel), two *roamer* dogs (RR kernel), a *stay-at-home* dog and a *roamer* dog (SR kernel) and two *stay-at-home* dogs (SS kernel).

### Model simulations

The six spatial kernel scenarios produced significantly different model outputs within each of the index dog scenarios (KW test *P* < 0.001 for both outbreak duration and number of rabid dogs; Table 3). The kernels that represent contact with at least one *stay-at-home* dog (SS, SR and ES kernels) predicted potential outbreaks with the fewest rabid dogs in all three index dog scenarios (Table 3 and Supplementary material 1). The smallest was produced by the SS kernel with an index dog in a sparse area (10 dogs, range = 1–453) which represents 1.23% (0.12–55.72%) of the NPA dog population. Conversely, outbreaks that caused the most rabid dogs were consistently produced by the EE kernel in all index dog scenarios; the largest median was an index dog in a dense area (698 dogs, range = 1–745 dogs; Table 3). This represents 85.9% (0.12–91.6%) of the entire NPA dog population.

**Table 3.** Model outputs and mean ranks (Dunn's Test) of 18 rabies-spread models utilising six spatial kernels to inform contacts in three index dog scenarios (dense, sparse and random)

Scenario^a^	Outbreak duration			Rabid dogs		
	Median (min–max days)	Mean rank	Pairwise comparison	Median (min–max dogs)	Mean rank	Pairwise comparison
Dense						
EE	423 (29–827)	3654	a	698 (1–745)	10 518	e
ER	499 (29–1028)	5441	b	645 (1–717)	8396	d
ES	609 (29–1440)	6971	c	460 (1–597)	4081	b
RR	562 (28–1090)	6802	c	606 (1–673)	6922	c
SR	649 (29–1529)	7649	d	449 (1–568)	3926	b
SS	433 (28–1580)	5486	b	84 (1–448)	2159	a
Sparse						
EE	455 (29–935)	5847	bc	692 (1–743)	8759	e
ER	519 (27–1056)	6523	d	635 (1–700)	7401	d
ES	155 (29–1491)	5583	b	11 (1–569)	4481	b
RR	556 (29–1351)	6988	e	582 (1–693)	6695	c
SR	231 (29–1526)	6190	cd	18 (1–568)	4782	b
SS	148 (29–1377)	4872	a	10 (1–435)	3885	a
Random						
EE	433 (28–782)	4859	a	697 (1–749)	10 121	e
ER	499 (28–1122)	6051	b	643 (1–700)	7959	d
ES	555 (29–1337)	6472	c	442 (1–584)	4329	b
RR	549 (28–1123)	6820	c	595 (1–694)	6619	c
SR	577 (29–1468)	6679	c	392 (1–568)	4088	b
SS	239 (28–1466)	5122	a	22 (1–441)	2887	a

Mean ranks with letters in common are not significantly different within index dog scenarios, *P* ⩾ 0.01.

a Spatial kernels based on all possible combinations of categories between a pair of dogs; two *explorer* dogs (EE kernel), an *explorer* dog and a *roamer* dog (ER kernel), an *explorer* dog and a *stay-at-home* dog (ES kernel), two *roamer* dogs (RR kernel), a *stay-at-home* dog and a *roamer* dog (SR kernel) and two *stay-at-home* dogs (SS kernel).

The longest median outbreak duration was produced by the SR kernel with an index dog in a dense area (649 days, range = 29–1529 days) followed by the ES kernel with an index dog in a dense area (609 days, range = 29–1440; Table 3 and Supplementary material 2). The shortest median duration was produced by the SS kernel with an index dog in a sparse area (148 days, range = 29–1377 days). The SR and ES kernel also produced the longest outbreak durations with a random index dog. However, with an index dog in a sparse area, these two kernels produced shorter outbreak durations compared with the other kernels, except the SS kernel.

**Fig. 3.**
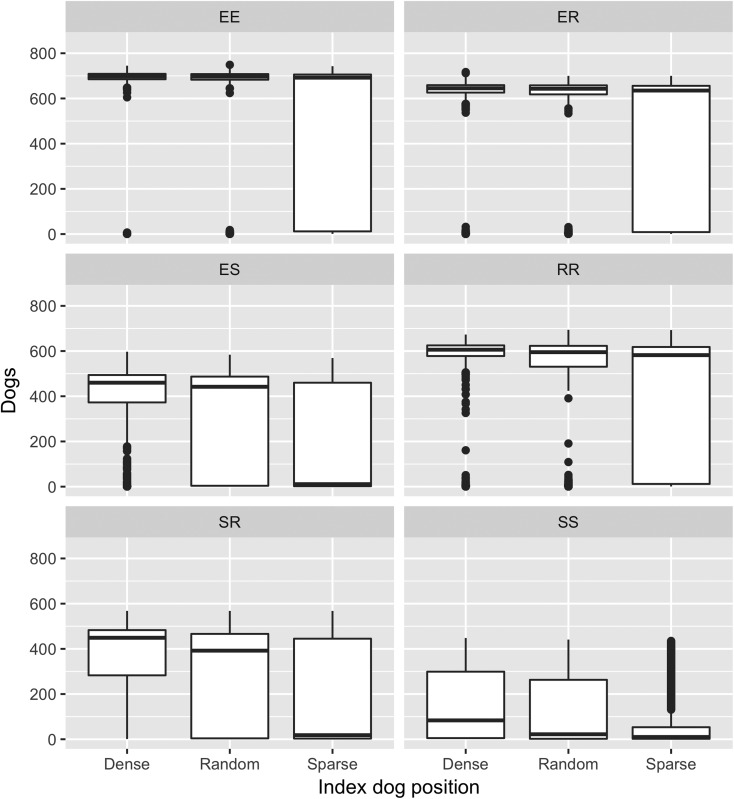
Boxplots of the number of rabid dogs predicted with three index dog scenarios and six spatial kernels in a rabies spread model in the Northern Peninsular Area. Boxplots with letters in common have mean ranks that are not significantly different, Dunn's Test *P* ⩾ 0.01. Spatial kernels: two *explorer* dogs (EE kernel), an *explorer* dog and a *roamer* dog (ER kernel), an *explorer* dog and a *stay-at-home* dog (ES kernel), two *roamer* dogs (RR kernel), a *stay-at-home* dog and a *roamer* dog (SR kernel) and two *stay-at-home* dogs (SS kernel).

**Fig. 4.**
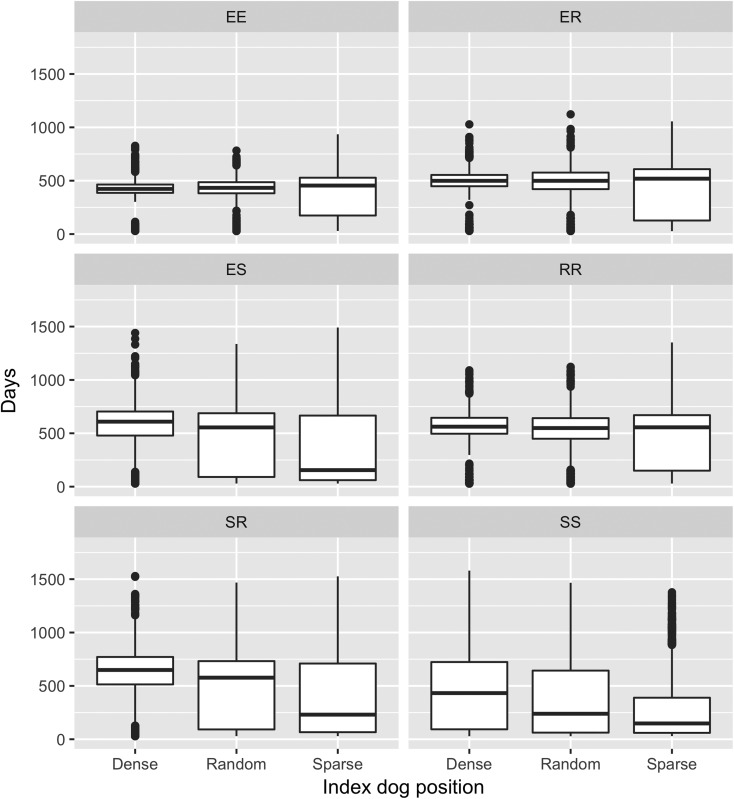
Boxplots of outbreak duration predicted with three index dog scenarios and six spatial kernels. Boxplots with letters in common have mean ranks that are not significantly different, Dunn's Test *P* ⩾ 0.01. Spatial kernels: two *explorer* dogs (EE kernel), an *explorer* dog and a *roamer* dog (ER kernel), an *explorer* dog and a *stay-at-home* dog (ES kernel), two *roamer* dogs (RR kernel), a *stay-at-home* dog and a *roamer* dog (SR kernel) and two *stay-at-home* dogs (SS kernel).

The Kruskal–Wallis test demonstrated significant differences between outbreak duration for all scenarios except the ER kernel (*P* = 0.27). The dense index dog scenarios produced outbreaks that resulted in significantly larger numbers of rabid dogs in all kernel scenarios and the sparse index dog resulted in significantly fewer numbers of rabid dogs in all kernel scenarios (Fig. 3). The position of the index dog did not have as great an effect on outbreak duration for the EE, ER and RR kernels; there were multiple non-significant pairwise (Dunn's Test) comparisons within these kernels (Fig. 4). For example, the ER kernels produced much more similar outbreak duration medians of 499, 519 and 499 for an index dog in a dense area, a sparse area and a randomly positioned index dog, respectively, compared with the varied SS kernel outbreak duration medians of 433, 148 and 239 days for an index dog in a dense area, a sparse area and a randomly positioned index dog, respectively.

### Model convergence

The simulation number at which 97.5% of CVs in the previous 100 simulations were <0.04 for outbreak duration was 1000. However, this was reached at 2000 simulations for the number of rabid dogs as the outcome of interest. Therefore, 2000 iterations were considered sufficient to produce converged outputs for both outbreak duration and number of rabid dogs from all simulations.

## Discussion

The PHR overlap index was used to create six different spatial kernels to describe the heterogeneous contacts between dogs of the same and different roaming categories, which were previously characterised based on field GPS data [10]. Using these six spatial kernels, different rabies outbreaks in the NPA dog population were predicted with respect to outbreak duration and number of rabid dogs.

The underlying roaming behaviours of the dogs determined the shape of the spatial kernels and subsequently the number of contacts and effective contacts and the type of outbreak for each kernel simulation. The relatively lower probability of contact at shorter distances of the EE, ER and ES kernels is because the time density of *explorer* dogs’ UD is generally lower around their residence since the area of their 50% HR isopleth is greater compared with the dogs in other roaming categories [10]. When the second dog in the contact pair is another *explorer* dog or a *roamer* dog (EE and ER kernel) the maximum distances at which contact was possible was large (1000 and 881 m for the EE and ER kernels, respectively; Table 2) because both dogs in the pair have large HRs facilitating a probability of contact when their residences are far apart. Consequently, the number of contacts and subsequent effective contacts associated with these kernels are greater than the other kernels, causing outbreaks with a higher number of rabid dogs but over a shorter time period. This is a similar finding to a study in which modelling predicted a rabies incursion to cause total population decline during a short time period in a high-contact, free-roaming dog population in an Australian Indigenous community, compared with a relatively unaffected peri-urban location in which fewer effective contacts occurred between the roaming dogs [8].

When the second dog is a *stay-at-home* dog (ES kernel) – which spends almost all its time at or around its residence – the kernel also has low contact probabilities at shorter distances between residences like the EE and ER kernels, but the maximum distance at which a contact can occur is shorter. This is because the *stay-at-home* dog does not have a large HR nor does it regularly roam to contact an *explorer* dog far from its residence. Therefore, the overall number of contacts is much lower than the EE and ER kernels and results in smaller outbreaks that last longer due to slower spread of rabies.

Conversely, the SR kernel has high contact probabilities at short distances between residences because the two dogs in the contacting pair spend most of their time at their residences at a short distance apart; this results in a high degree of UD overlap for short distances between their residences. The contact probability declines quickly at longer distances, again because the dogs tend to stay at home and consequently, the UD overlap is less. Therefore, the number of contacts are few, causing slow disease spread and subsequently longer outbreaks. This is similar for the SS kernel. However, both dogs in the contacting pair remain around their residence, which causes overall so few number of effective contacts that often outbreaks did not develop. Subsequently, the SS kernel produced the shortest median outbreak duration and few predicted rabid dogs. These results are similar to those in a model of wild dogs in Australia, in which a small area traversed per day (i.e. low sociability and therefore fewer individual contacts) caused a slower spread of rabies within the population and often led to a low probability of outbreak propagation [7].

Although the RR kernel has a similar shape to the SS and SR kernels (Fig. 2), the overall number of contacts − and therefore predicted outbreaks − were more similar to the EE and ER outbreaks with shorter duration and more rabid dogs than outbreaks with the SS and SR kernel outbreak. Both dogs in the RR contacting pair spend most of their time at home so there is high probability of contact at shorter distances. Also, both dogs roam from their residence so there is a longer maximum distance for contact probabilities compared with the SS and SR kernels. Previous studies have suggested that dogs that have larger HRs could have an important influence on the spread of epidemics, especially rabies [10, 11, 23, 24]. This is reflected in this study by the EE outbreaks. However, this study also highlights the importance of the mid-ranging dogs (described as *roamers*) and the combination of the two in the spread of epidemics (the ER and RR outbreaks).

The three roaming categories co-exist in the NPA population [10], creating heterogeneous probabilities of contact in the population. The proportion of dogs in each roaming category in the NPA population could have an important influence on the duration and spread of potential rabies outbreaks in the area. If there were a high proportion of *explorer* dogs, there would likely be short outbreaks with a high number of rabid dogs. Conversely, if the proportion of *stay-at-home* dogs was high, the outbreaks would tend to be of longer duration but with fewer rabid dogs. The model predictions in this study represent extreme possibilities for types of rabies outbreaks in the NPA because they assume only one type of contact probability (e.g. all contacts governed by the EE kernel). To accurately model the complex contact structure of the NPA population, dogs would need to be assigned to the three roaming categories so that potential contact probabilities can be modelled using all six kernels in one model, dependent on the roaming categories of the dogs. Also, there is evidence of interaction between dingoes and domestic free-roaming dogs in the NPA [25], and therefore, the proportion of dogs in each category could affect the probability of rabies spill-over into surrounding wildlife, especially wild dogs and dingoes, which could extend a potential rabies outbreak. If there are more *stay-at-home* dogs, the probability of spill-over would likely be lower than if there were more *explorer* dogs. However, the proportion of dogs in each roaming category for the NPA dog population is currently unknown.

The PHR index was considered the most valid overlap index to estimate the probability of contact between pairs of dogs. It was preferred to the HR index (which provides similar information – the probability of finding dog *i* within dog *j*’s HR and *vice versa*) because the HR index is only based on area of overlap and does not consider the time distribution within the HR area that is provided by the UD [17]. Other UD overlap indices − such as the volume index (VI) and utilisation distribution overlap index (UDOI) [17] − generate one number describing the degree of UD overlap or similarity (usually 0 representing no overlap or similarity and 1 complete overlap). These have been used in previous studies mainly to understand shared-space use or UD similarities of various animals for population conservation or management purposes [26–29]. When comparing sets of UD overlaps, the UDOI and VI have better discriminatory power in a set of paired examples over the PHR (see Example II in Fieberg and Kochanny [17]) and are the recommended indices to use when the aim is to quantify the degree of similarity among UD estimates or quantify space-use sharing [17–18]. However, the aim of the current study was to estimate contact probabilities using overlapping UDs − not to compare overlaps. Unlike the PHR index − which is a direct estimation of probability of finding dogs in other dog's UDs − UDOI and VI cannot be easily converted to probability of contacts.

A key aspect of this study was the use of UD overlap and the PHR index to inform the probability of contact between pairs of dogs [17]. Although this could over or underestimate the probability of contact for individual pairs of dogs – for example, pairs of dogs could actively avoid or seek each other's company – there is evidence that UD overlap is significantly correlated to contact rate [18]. In addition, the distributions of individual dogs’ UDs are likely to vary throughout each 24 h period; therefore, a pair of dogs might visit their potential overlap area at different times. However, given that the model simulates a population of dogs which are likely to exhibit a range of social behaviours, uses a daily time step, and the findings of Robert *et al*. [18], the use of UD overlap and the PHR index provide a useful proxy of the daily probability of contact when data that could directly estimate the contact rate are lacking. Examples of such field data include video cameras on dogs that show if a contact sufficient for rabies transmission occurred [15], ultra-high-frequency proximity loggers that directly record contacts between studied individuals [30], or GPS recording devices on dogs with locations recorded at intervals sufficient to infer spatiotemporal association between a pair of dogs [9, 11, 15, 16, 25]. Although field data that directly estimate contact rates might be more accurate, they are often expensive, labour intensive and subject to bias.

A limitation of this study was the relatively poor goodness of fit of the data to the three functions assessed for predicting the probability of contact at 1 m increments, and generalizing the data to the NPA dog population. The main reason for the poorer fits was variable contact probabilities at longer distances (600–1000 m); *explorers* roam far and to various places and *roamers* irregularly roam. The datasets were also likely subject to selection bias. There is a possibility that dogs who are far-roaming (*explorer* dogs) are less likely to be collared in the field (less accessible) or that their collars are more likely to be lost or damaged. The sample size of *explorer* and *roamer* dogs was lower than *stay-at-home* dogs (*n* = 6 for *explorers* and *roamers* and *n* = 9 for *stay-at-home*). Consequently, there were fewer UDs that overlapped at long distances, and these were less predictable. Goodness of fit could be improved by targeting *explorer* and *roamer* dogs to collect additional field data to capture more long-distance roaming and more accurately define the edges of their UDs.

The field datasets collected by Hudson *et al*. [10] consisted of only 21 dogs, out of an estimated population of 813 dogs [6]. Thus, there is a potential lack of representativeness caused by the relatively small sample size. The PHR index accounts for time distribution within the HR area so variation in UD overlap corresponds to different roaming behaviours within the HR. Therefore, simulating different orientations of pairs of UDs and their resulting overlap helped capture the maximum variation in roaming behaviours within this restricted sample size. Also, the small sample size restricts investigation into other variables such as sex, age and other social parameters that could directly influence contact rates and indirectly affect contacts through changed roaming patterns. More field data would be required to investigate which dogs belong in each category or if dog traits determine roaming category.

Finally, the dog density surrounding the selected index dog had an effect on the model outputs because of the underlying structure of the agent-based spatial simulation model [9]. This could subsequently confound the results, i.e. differences in the predicted outbreaks are due to differences in dog density and not the kernels that were used. Although the outbreaks produced for each kernel within each index dog scenario were often significantly different for outbreak duration and number of rabid dogs (Figs 3 and 4), the influence of the kernels on the outbreaks remained the same in each index dog scenario, but on different scales. For example, the EE kernel always produced short outbreaks with the greatest number of rabid dogs in all scenarios, regardless of dog density. The exception was the SR and ES kernels, which produced long outbreaks with few rabid dogs in the random and dense index dog scenarios but in the sparse scenario, these kernels produce short outbreaks with few rabid dogs. This suggests that if the dog density surrounding the index dog is sparse, the probability of an outbreak occurring is lower when using SR and ES kernels.

The spatial kernels created in this study describe the heterogeneous contacts within and between recently defined roaming categories in the NPA dog population. Once incorporated into a rabies-spread model for the NPA, the spatial kernels produced a significant effect on rabies spread which was dependent on the underlying roaming behaviours of the dogs. An improved understanding of the complex contact structure of the roaming categories in the NPA dogs and their effect on rabies spread allows the investigation and development of best practice control strategies to help mitigate the risk of a rabies outbreak. Vaccination is central to programmes to control and eliminate rabies in many regions; a vaccination coverage rate of 70% or higher has been recommended to achieve control [2, 4, 31, 32]. However, this goal can be difficult to achieve in regions that lack resources and infrastructure [33]. In such situations, targeted control strategies might be more economic and the incorporation of the roaming category contact kernels into the rabies model allows future exploration of such targeted vaccination strategies. Future research could extend current knowledge on risk factors of roaming by investigating dog traits that correlate to roaming category so that such targeted strategies could be better implemented. For example, targeting only the *explorer* dogs for vaccination because of their effects on rabies propagation might be more efficient than a random vaccination approach, even if the vaccination coverage is lower. However, the logistical and economic benefits need to be investigated using field data.

## Supplementary material

For supplementary material accompanying this paper visit https://doi.org/10.1017/S0950268819000189. click here to view supplementary material
