# Supplementary material for: Using roaming behaviours of dogs to estimate contact rates: the predicted effect on rabies spread
Source: Epidemiol Infect. 2019 Mar 5;147:e135. doi: 10.1017/S0950268819000189 (PMC6518777; doi:10.1017/S0950268819000189)
Supplement: Supplementary file 1 [file S0950268819000189sup001.zip › Supplementary_Figure_legends_c.docx]

Supplementary Figure 1: Boxplots of the number of rabid dogs produced by six spatial kernels in three index dog scenarios used to describe the potential rabies spread in the Northern Peninsular Area. Spatial kernel: two *explorer* dogs (EE kernel), an *explorer* dog and a *roamer* dog (ER kernel), an *explorer* dog and a *stay-at-home* dog (ES kernel), two *roamer* dogs (RR kernel), a *stay-at-home* dog and a *roamer* dog (SR kernel) and two *stay-at-home* dogs (SS kernel).

Supplementary Figure 2: Boxplots of the outbreak durations produced by six spatial kernels in three index dog scenarios used to describe the potential rabies spread in the Northern Peninsular Area. Spatial kernel: two *explorer* dogs (EE kernel), an *explorer* dog and a *roamer* dog (ER kernel), an *explorer* dog and a *stay-at-home* dog (ES kernel), two *roamer* dogs (RR kernel), a *stay-at-home* dog and a *roamer* dog (SR kernel) and two *stay-at-home* dogs (SS kernel)
