# Supplementary figures and images for: Using roaming behaviours of dogs to estimate contact rates: the predicted effect on rabies spread
Source: Epidemiol Infect. 2019 Mar 5;147:e135. doi: 10.1017/S0950268819000189 (PMC6518777; doi:10.1017/S0950268819000189)

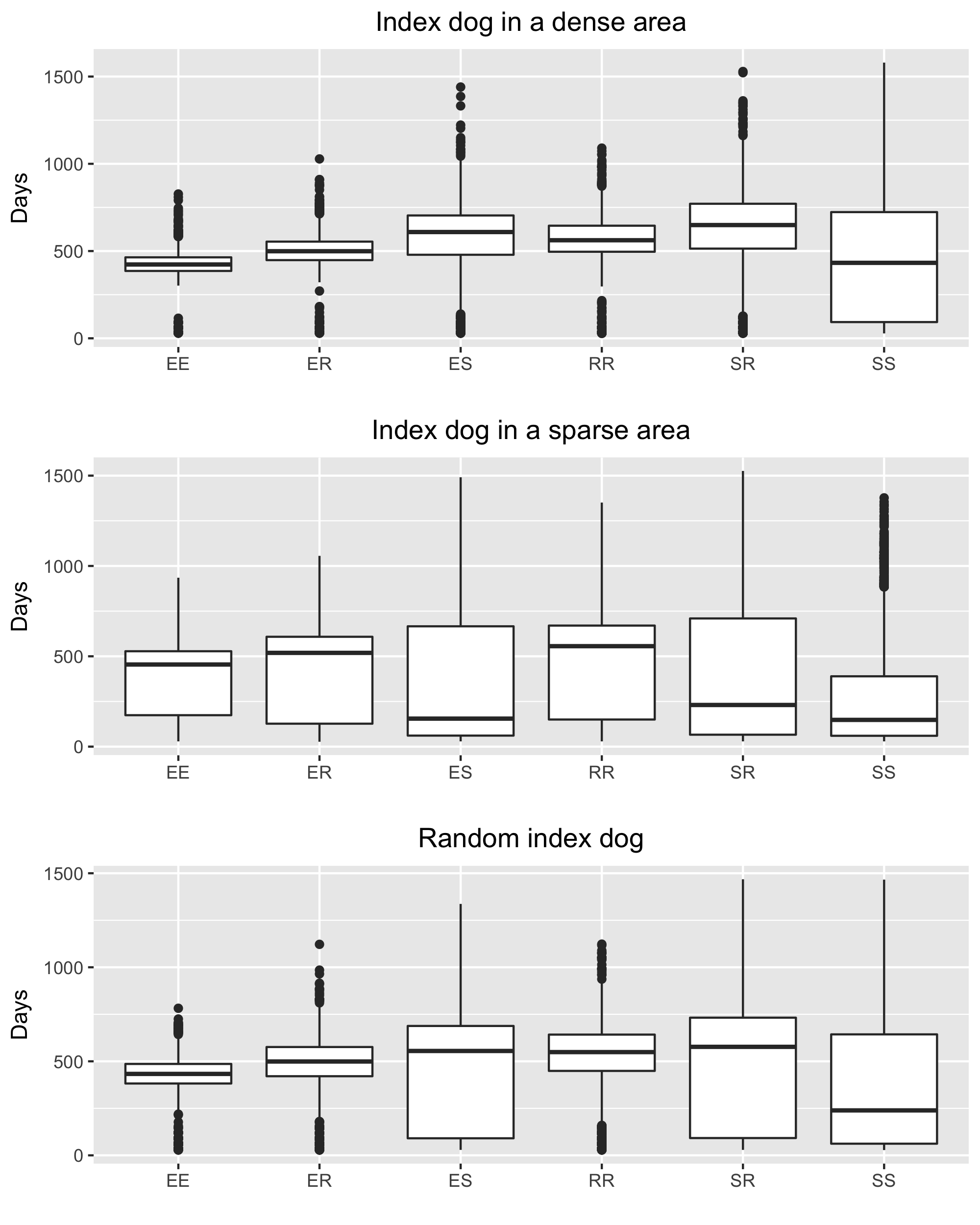

Supplement: Supplementary file 1 [file S0950268819000189sup001.zip › SUPP2.tiff]

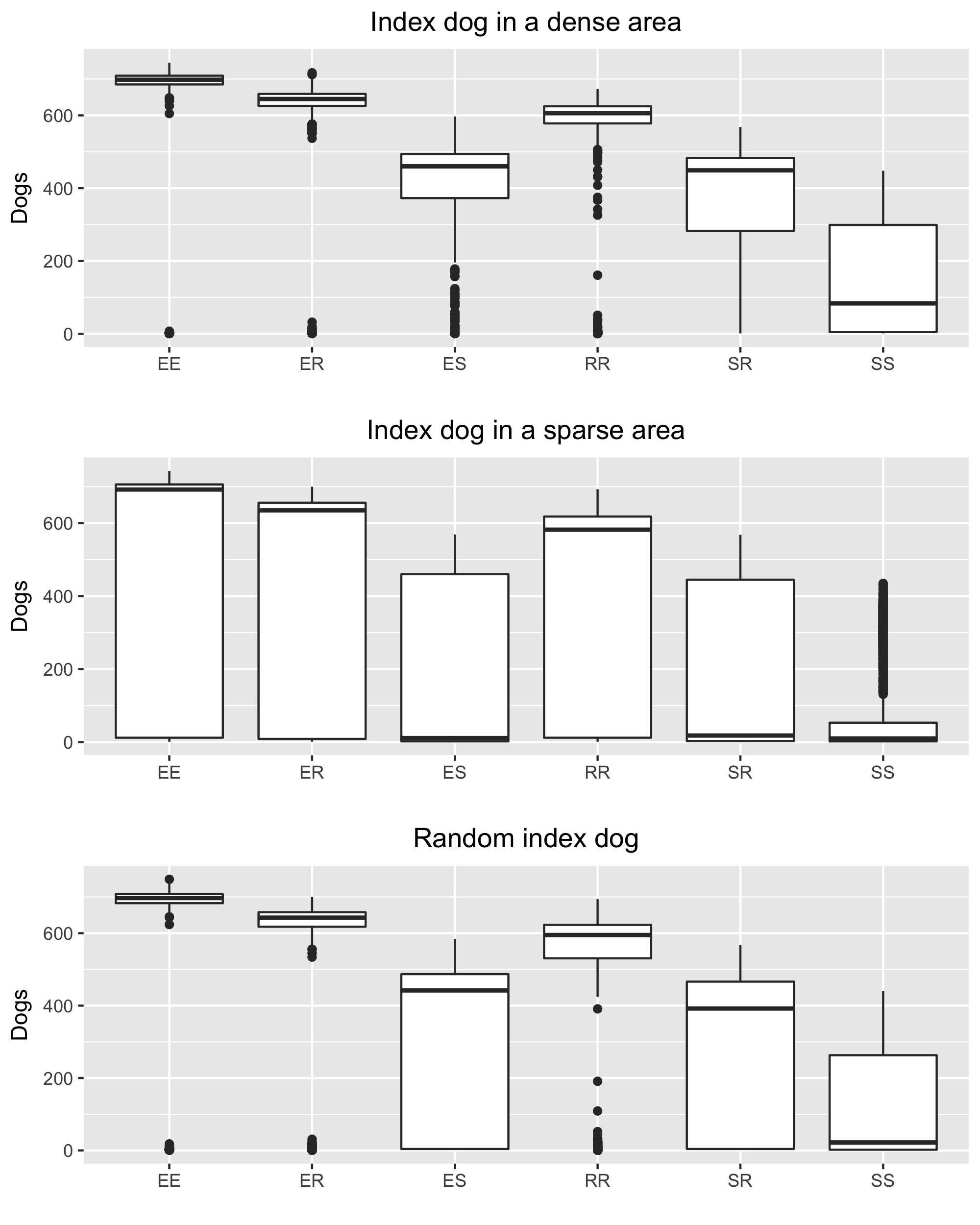

Supplement: Supplementary file 1 [file S0950268819000189sup001.zip › SUPP1.tiff]
